# New Spectrofluorimetric Method with Enhanced Sensitivity for Determination of Paroxetine in Dosage Forms and Plasma

**DOI:** 10.4137/aci.s1053

**Published:** 2008-11-18

**Authors:** Ibrahim A. Darwish, Sawsan M. Amer, Heba H. Abdine, Lama I. Al-Rayes

**Affiliations:** Department of Pharmaceutical Chemistry, College of Pharmacy, King Saud University, P.O. Box 2457, Riyadh 11451, Saudi Arabia

**Keywords:** paroxetine, NBD-C, spectroflourimetry, pharmaceutical analysis

## Abstract

New simple spectrofluorimetric method with enhanced sensitivity has been developed and validated for the determination of the antidepressant paroxetine (PXT) in its dosage forms and plasma. The method was based on nucleophilic substitution reaction of PXT with 4-chloro-7-nitrobenzo-2-oxa-1,3-diazole in an alkaline medium (pH 8) to form a highly fluorescent derivative that was measured at 545 nm after excitation at 490 nm. The factors affecting the reaction was carefully studied and optimized. The kinetics of the reaction was investigated, and the reaction mechanism was presented. Under the optimized conditions, linear relationship with good correlation coefficient (0.9993) was found between the fluorescence intensity and PXT concentration in the range of 80–800 ng ml^−1^. The limits of detection and quantitation for the method were 25 and 77 ng ml^−1^, respectively. The precision of the method was satisfactory; the values of relative standard deviations did not exceed 3%. The proposed method was successfully applied to the determination of PXT in its pharmaceutical tablets with good accuracy; the recovery values were 100.2 ± 1.61%. The results obtained by the proposed method were comparable with those obtained by the official method. The proposed method is superior to the previously reported spectrofluorimetric method for determination of PXT in terms of its higher sensitivity and wider linear range. The high sensitivity of the method allowed its successful application to the analysis of PXT in spiked human plasma. The proposed method is practical and valuable for its routine application in quality control and clinical laboratories for analysis of PXT.

## Introduction

Paroxetine (PXT) is a new generation antidepressant drug. It exerts its antidepressant effect through a selective inhibition for the reuptake of the neurotransmitter serotonin by the presynaptic receptors. PXT is comparable to the tricyclic antidepressants in their clinical efficacy, however PXT is safer and has greater acceptance by the patients [1]. It is also prescribed in the treatment of related disorders, such as obsessive-compulsive disorder, panic fits, social phobia, and post-traumatic stress [2]. PXT is devoid of sedative effect and remarkably safe in overdose. PXT takes 5.2 hours to reach the peak, with extended half-life (21 hours) that allowed the introduction of formulations for once-daily dosing [3]. These combined qualities made PXT the most widely prescribed antidepressants [4].

The methods reported for quantitative determination of PXT in dosage forms and/or biological fluids include voltammetry [5,6], densitometry [7,8], high performance liquid chromatography [9–14], gas chromatography [15–17], capillary electrophoresis [18], spectrophotometry [19–21], and spectrofluorimetry [22]. The chromatographic and electrophoretic methods offered the required sensitivity and selectivity for the analysis of PXT in biological fluids, however their sophisticated instrumentation, tedious sample clean-up procedures, and high analysis cost limited their routine use for determination of PXT in quality control and clinical laboratories. Spectrophotometric methods, although the inherent simplicity of the technique, were time-consuming as they involved laborious multiple extraction steps and long reaction time [21], and they had inadequate sensitivity for determination of PXT in biological fluids.

Spectrofluorimetry is considered a sensitive and simple technique. Only one spectrofluorimetric method has been described for determination of PXT based on measuring its native fluorescence [22], with limit of quantitation of 50 ng ml^−1^. Although the authors claimed the applicability of the method for determination of PXT in spiked human plasma based on the recovery of spiked PXT, however the spiked concentrations (100–400 ng ml^−1^) were higher than the reported maximum plasma level of PXT (10–30 ng ml^−1^) [23], in addition to the poor recovery (77.7%) obtained by this method. For these reasons, the development of new alternative more sensitive spectrofluorimetric method for the determination of PXT was very essential.

The present study describes the development and validation of a new simple spectrofluorimetric method with enhanced sensitivity for determination of PXT in dosage forms and plasma with a limit of detection of 25 ng ml^−1^. The method was based on the derivatization of PXT with 4-chloro-7-nitobenzofurazan (NBD-Cl) reagent to produce a fluorescent derivative. The induced fluorescence intensity was measured at 545 nm after excitation at 490 nm, and correlated with the PXT concentrations.

## Experimental

### Apparatus

FP-6200 fluorometer (JASCO Co. Ltd., Kyoto, Japan), with 1-cm quartz cells was used for all measurements. The slit width of both the excitation and emission monochromators was set at 1.5 nm. The calibration and linearity of the instrument were frequently checked with standard quinine sulphate (0.01 μg ml^−1^). Wavelength calibration was performed by measuring λ_excitation_ 275 nm and λ_emission_ 430 nm; no variation in the wavelength was observed. pH meter, Model 350 (Bibby Scientific Ltd., T/As Jenway, Essex, England). MLW type thermostatically controlled water bath (Memmert GmbH, Co. Schwa bach, Germany).

### Reagents and materials

Paroxetine hydrochloride (PXT; SmithKline Beecham Pharmaceuticals, Bentford, England) was obtained and used as received; its purity was 99.8 ± 1.45%. A solution of 0.2% (w/v) of 4-chloro-7-nitrobenzo-2-oxa-1,3-diazole (NBD-Cl; Sigma Chemical Co., St. Louis, U.S.A.) was freshly prepared by dissolving 100 mg in 50 ml acetone. Clark and Lubs buffer solution of pH 8 was prepared by mixing 50 ml of 0.2 M aqueous solution of boric acid and KCl (1 liter contains 12.368 g of boric acid and 14.90 g of KCl) with 0.2 M NaOH in 200 ml standard flask [24], and adjusted by pH meter. Seroxate tablets (SmithKline Beecham Pharmaceuticals) are labeled to contain 20 mg PXT per tablet. Human plasma samples were collected from normal healthy volunteer at King Khaled University Hospital (Riyadh, Saudi Arabia), and they were stored in deep-freezer at −20 °C until analysis. Double distilled water was obtained through WSC-85 water purification system (Hamilton Laboratory Glass Ltd., Kent, U.S.A.), and used throughout the work. All solvents and materials used throughout this study were of analytical grade.

### Preparation of standard and sample solutions

#### Paroxetine hydrochloride (PXT) standard solution

An accurately weighed amount (50 mg) of PXT was quantitatively transferred into a 25-ml calibrated flask, dissolved in 20 ml distilled water, completed to volume with the same solvent to obtain a stock solution of 2 mg ml^−1^. This stock solution was further diluted with water to obtain working solutions in the ranges of 800–8000 ng ml^−1^.

#### Tablets sample solution

Twenty tablets were weighed, and finely powdered. An accurately weighed quantity of the powdered tablets equivalent to 100 mg of PXT was transferred into a 100-ml calibrated flask, and dissolved in about 40 ml of distilled water. The contents of the flask were swirled, sonicated for 5 min, and then completed to volume with water. The contents were mixed well and filtered rejecting the first portion of the filtrate. The prepared solution was diluted quantitatively with distilled water to obtain a suitable concentration for the analysis.

#### Plasma samples

Aliquots of 1 ml of PXT solution (800–8000 ng ml^−1^) were dispensed into test tubes. A 1 ml of drug-free plasma and 2 ml of acetonitrile was added. After vortexing for 3 min, the tube was centrifuged at 4500 rpm for 20 min. The mixture was rendered alkaline with 0.1 M NaOH and then extracted three times with 3 × 1.5 ml hexane:isoamyl alcohol (99:1, v/v). The extract was transferred to another 5 ml tube and evaporated to dryness under stream of nitrogen gas at 40 °C. The residue was dissolved in 1 ml acetone and used for analysis.

### General recommended procedure

Accurately measured aliquots of PXT solution containing 800–8000 ng ml^−1^ were transferred into separate 10-ml calibrated flasks. One milliliter of Clark and Lubs buffer solution (pH 8 ± 0.2) was added followed by 1 ml of NBD-Cl solution (0.2%, w/v). The reaction solution was allowed to proceed at 40 ± 5 °C for 30 min. After cooling, the reaction mixture was acidified by adding 1 ml of 0.1 M HCl, and completed to volume with acetone. The fluorescence intensity of the resulting solution was measured at 490 nm after excitation at 545 nm against reagent blanks prepared in the same manner with 1 ml water instead of 1 ml sample solution.

### Determination of the stoichiometric ratio of the reaction

In the limiting logarithmic method [25], two sets of experiments were carried out employing the general recommended procedures described above. The first set of experiments were carried using increasing NBD-Cl concentrations (5 × 10^−5^–5 × 10^−4^ M) at fixed PXT concentration (1.37 × 10^−6^ M). The second set of experiments were carried using increasing PXT concentrations (3.28 × 10^−7^–2.19 × 10^−6^ M) at fixed NBD-Cl concentration (1 × 10^−3^ M). The logarithms of the obtained fluorescence intensities were plotted as a function of the logarithms of the NBD-Cl and PXT concentration in the first and second sets of experiments, respectively. The slopes of the fitting lines in both sets of experiments were calculated.

## Results and Discussion

### Strategy for assay development and spectral characteristics

**Figure 1:**
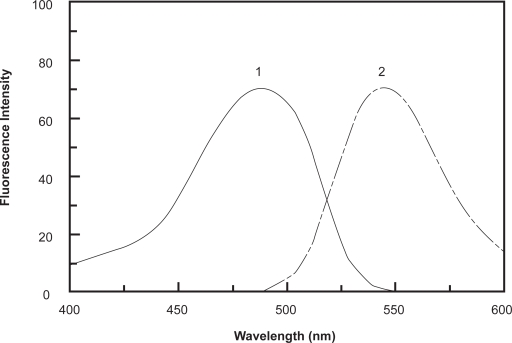
Excitation (1) and emission (2) spectra of the reaction product of PXT (300 ng ml^−1^) with NBD-Cl (0.2%, w/v).

PXT exhibits weak native fluorescence, thus its derivatization with fluorogenic reagent was necessary to increase its detectability and ultimately the sensitivity for its spectrofluorimetric determination. NBD-Cl has been used as a derivatizing reagent in the spectrofluorimetric determination of many pharmaceutical amines [26–29]. It forms highly fluorescent derivatives with amines using relatively mild reaction conditions. The reaction of NBD-Cl with PXT has not been investigated yet. Therefore, the present study was devoted to investigate the reaction between NBD-Cl and PXT (being amine), and employment the reaction in the development of sensitive and simple spectrofluorimetric method for determination of PXT in dosage forms and plasma. Owing to the presence of labile chloride in the chemical structure of NBD-Cl, a daily fresh solution was prepared and tested in the present study. It was found that PXT reacts with NBD-Cl and forms a yellow fluorescent derivative. This derivative exhibited maximum fluorescence intensity (λ_em_) at 545 nm after its excitation at maximum wavelength (λ_ex_) at 490 nm. The excitation and emission spectra for the reaction product of PXT with NBD-Cl are given in Figure. 1.

### Optimization of reaction variables

#### Effect of NBD-Cl Concentration

**Figure 2:**
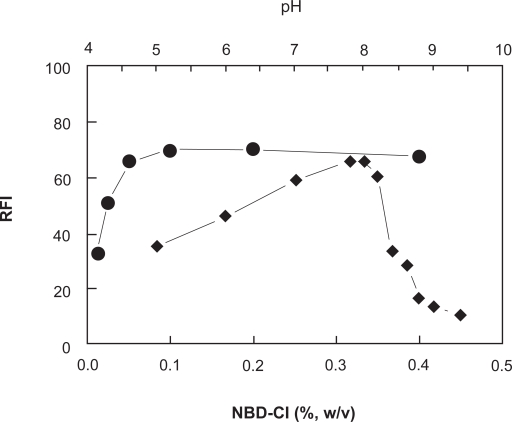
Effect of NBD-Cl concentration (•) and pH (◆) on the fluorescence intensity of the product of the reaction of PXT (600 ng ml ^−1^) and NBD-Cl.

The effect of NBD-Cl concentration on its reaction with PXT revealed that the reaction was dependent on NBD-Cl concentration as the fluorescence intensity (FI) of the reaction solution increased as the NBD-Cl concentration increased (Fig. 2). The highest readings were attained at NBD-Cl concentration of 0.1% (w/v). At higher concentrations of NBD-Cl up to 0.4%, the fluorescence intensity was not affected. For more precise readings, further experiments were carried out using 0.2%.

#### Effect of temperature and time

**Figure 3:**
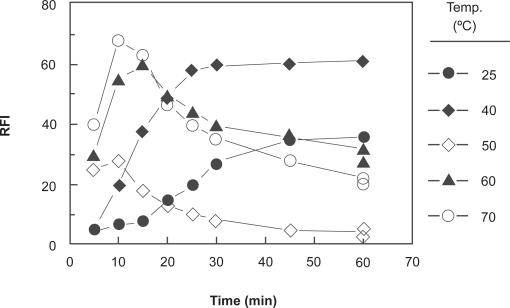
Effect of time on the reaction of NBD-Cl (0.2%, w/v) with PXT (600 ng ml^−1^) at different temperatures. RFI is the relative fluorescence intensity.

In order to determine the optimum temperature and time required for completion the reaction, the derivatization reaction was carried out at room temperature (25 ± 5 °C) and the induced FI values were monitored at different time intervals. It was found that the reaction was very slow, and did not go to completion in reasonable time; it required more than 1 hour (Fig. 3). Therefore, investigations were carried out at varying elevated temperatures (40–70 °C), and the intensities of the induced fluorescence were monitored for 60 min. The optimum conditions were considered as the conditions at which the high FI values, high reproducible results, and comfortable measurements (wide plateau region on the FI-time curve) could be obtained. The results indicated that the reaction was dependent on temperature, and the optimum condition was achieved by heating at 40 °C for 30 min. At higher temperatures, the maximum FI was obtained in shorter times (∼10 min), however rapid progressive decrease in the readings was observed as the reaction time increased. This was probably attributed to the degradation of the reagent at high temperature. This observation was coincident with the results that have been previously reported by Aktas et al. [30].

#### Effect of pH

In order to generate the nucleophile from PXT, the reaction should be carried out in alkaline medium. Different alkaline buffer systems (borate, phosphate, and carbonate) having the same pH value (8) were tested. The highest FI was obtained when the reaction was carried out in Clark and Lubs buffer. With other buffers, either precipitation of white colloid occurred upon addition of NBD-Cl reagent solution, non reproducible results, and/or weak sensitivities were observed.

The effect of pH on the reaction was studied by carrying out the reaction in Clark and Lubs buffer solution of pH 5–9.5. The results indicated that the FI increased as the pH increased and maximum readings were obtained at pH 8 ± 0.2 (Fig. 2). This increase in the FI with the pH was possibly due to the conversion of amino group of PXT from the HCl salt form (in acidic pH values) to the free amino group as the pH turns alkaline. This facilitates the nucleophilic substitution reaction. At pH above 8.2, sharp decrease in the readings occurred. This was attributed probably to the increase in the amount of hydroxide ion that holds back the condensation reaction between PXT and NBD-Cl. In order to keep the high sensitivity for determination of PXT, the subsequent experiments were carried out at pH 8 ± 0.2.

#### Effect of HCl concentration

Under the above mentioned conditions, significantly high fluorescence backgrounds were also observed. This was attributed to the hydrolysis of NBD-Cl to the corresponding hydroxy derivative namely, 4-hydroxy-7-nitrobenzo-2-oxa-1,3-diazole (NBD-OH) [31]. The fluorescence of NBD-OH was found to be quenched by decreasing the pH of the reaction medium to less than one [32]. Therefore acidification of the reaction mixture prior to measurement of the FI was necessary to remarkably decrease the background fluorescence. Meanwhile, the reaction product was not affected, thus the sensitivity was ultimately increased. The concentration of HCl required for acidification was found to be 0.01 M in the final assay solutions (i.e. 1 ml of 0.1 M).

#### Effect of diluting solvent

Upon diluting the reaction with water, colloids were obtained indicating the incomplete solubility of PXT-NBD in water. Therefore, water could not be used for dilution. In order to select the most appropriate organic solvent for diluting the reaction solution, different solvents were tested: methanol, ethanol, isopropanol, acetone, and acetonitrile. The highest readings were obtained when acetone was used. Therefore, acetone was used for diluting the reaction mixture in the subsequent experiments.

#### Stability of the fluorescent derivative

The effect of time on the stability of the PXT-NBD fluorescent derivative was studied by following the FI of the reaction solution (after dilution) at different time intervals. It was found that the FI values remain constant for at least 1 hour. This allowed the processing of large batches of samples, and their comfortable measurements with convenience. This increased the convenience of the method as well as made it applicable for large number of samples.

A summary for the optimization of the variables affecting the reaction of PXT with NBD-Cl is given in Table 1.

### Stoichiometry and kinetics of the reaction

**Figure 4:**
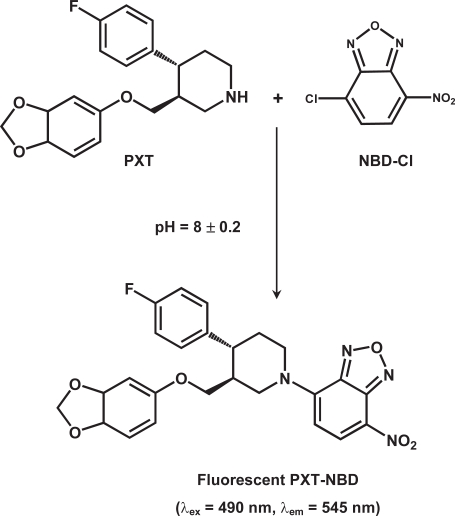
Scheme for the reaction pathway of PXT with NBD-Cl.

The stoichiometry of the reaction between PXT and NBD-Cl was investigated by limiting logarithmic method [25]. Two straight lines with comparable slopes (0.8035 and 0.9405) were obtained indicating the 1:1 ratio for the reactions. Based on this ratio, the reaction pathway between PXT and NBD-Cl was postulated to proceed as shown in Fig. 4.

Under the optimum conditions (Table 1), the fluorescence intensity-time curves for the reaction at varying PXT concentrations (2.73 × 10^−7^– 10.93 × 10^−7^ M) with a fixed concentration of NBD-Cl (1 × 10^−3^ M) were generated. The initial reaction rates (*k*) were determined from the slopes of these curves (from 0–25 min). The logarithms of the reaction rates (*Log k*) were plotted as a function of logarithms of PXT concentrations (*log C*). The regression analysis for the values was performed by fitting the data to the following equation:

$$Log\;k=log\;k^{\prime }+n\;log\;C$$

where k is reaction rate, *k*′ is the rate constant, *C* is the molar concentration of PXT, and *n* (slope of the regression line) is the order of the reaction. A straight line with slope values of 1.02527 (≈1) was obtained confirming that the reaction was first order. However under the optimized reaction conditions, the concentration of NBD-Cl was in much more excess than that of PXT in the reaction solution. Therefore, the reaction was regarded as a pseudo-first order reaction.

### The apparent rate constant and activation energy

**Figure 5:**
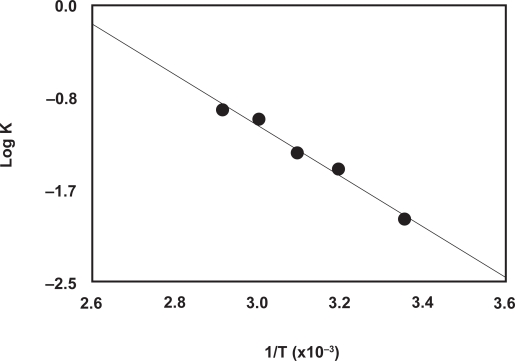
Arrhenius plot for the reaction of NBD-Cl and PXT. T and K are the absolute temperature and the apparent rate constant, respectively. [PXT] is 8.2 × 10^−7^ M and [NBD-Cl] is 1 × 10^−3^ M.

The fluorescence intensity-time curves at different temperatures (25, 40, 50, 60, and 70 °C) were generated using fixed concentration of PXT (8.2 × 10^−7^ M) and NBD-Cl (1 × 10^−3^ M). The reaction time was set at maximum 10 min, before the decrease of the FI occurs at high temperatures (Fig. 3). From these curves the apparent rate constants were calculated. The activation energy, defined as the minimum kinetic energy that a molecule possess in order to undergo a reaction, was determined using Arrhenius equation [33]:

Log k=log A−Ea/2.303 RT

where *k* is the apparent rate constant, *A* is the frequency factor, *Ea* is the activation energy, *T* is the absolute temperature (°C + 273), and *R* is the gas constant (1.987 calories degree^−1^ mole^−1^). The values of log *k* were plotted as a function of 1/*T*. Straight line with slope value of −2.2843 (= –*Ea*/2.303 *R*) was obtained (Fig. 5). From these data, the activation energy was calculated and found to be 43.73 *k* joule mole^−1^. This low activation energy explained that the nucleophilic substitution reactions between PXT and NBD-Cl could be easily takes place under mild conditions, and NBD-Cl could be used as a useful reagent in the spectrofluorimetric determination of PXT.

### Validation of the method

#### Calibration and sensitivity

**Figure 6:**
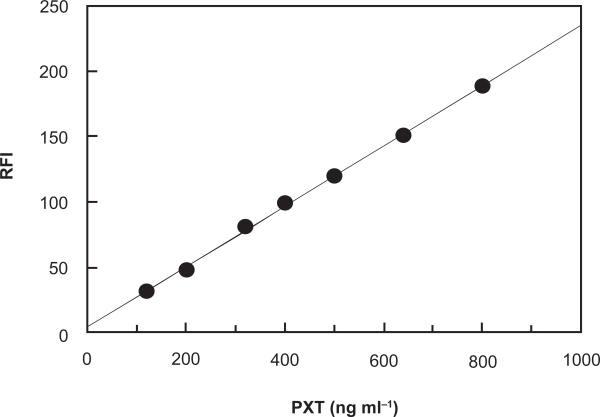
Calibration curve for spectrofluorimetric determination of PXT based on its reaction with NBD-Cl reagent. RFI is the relative fluorescence intensity.

Under the optimum conditions (Table 1), calibration curve for the determination of PXT by its reaction with NBD-Cl was constructed by plotting the FI as a function of the corresponding PXT concentration (Fig. 6). The regression equation for the results was: FI = a + b C, where FI is the fluorescence intensity, C is the concentration of PXT in ng ml^−1^. Linear relationship with small intercept and good correlation coefficient (0.9993) was obtained in the range of 80–800 ng ml^−1^. The limit of detection (LOD) and limit of quantitation (LOQ) were determined according to The International Conference of Harmonization (ICH) guidelines for validation of analytical procedures [34]. The LOD and LOQ values were found to be 25 and 77 ng ml^−1^, respectively. The parameters for the analytical performance of the proposed spectrofluorimetric method are summarized in Table 2.

#### Reproducibility

The reproducibility of the proposed method was determined by replicate analysis of five separate solution of the working standard. The method gave satisfactory results; RSD was 3% indicating its good reproducibility. This precision level is adequate for the precision and routine analysis of PXT.

#### Accuracy and specificity

The analytical recovery of the proposed spectrofluorimetric method was studied for added PXT concentrations, and the values were 97.8–101.2 ± 1.04–1.85% (Table 3), indicating the accuracy of the proposed method. The specificity of the method was evaluated by investigating the interference liabilities from the common excipients that might be added during pharmaceutical formulation. Samples were prepared by mixing known amount (20 mg) of PXT with various amounts of the common excipients: starch, glucose, lactose, acacia, talc, and magnesium stearate. These laboratory-prepared samples were analyzed by the proposed method applying he general 1 procedure. The recovery values were 98.76–103.02 ± 1.59%–2.15% (Table 4). These data confirmed the absence of interference from any of the common excipients with the determination of PXT by the proposed spectrofluorimetric method.

#### Robustness and ruggedness

Robustness was examined by evaluating the influence of small variation in the method variables on its analytical performance. In these experiments, one parameter was changed whereas the others were kept unchanged, and the recovery percentage was calculated each time. It was found that variation in the NBD-Cl concentrations (0.15%–0.25%, w/v), temperature (optimum ± 5 °C), and time (optimum ± 5 min) did not significantly affect the procedures; recovery values were 95.5–102.4, and the RSD values did not exceed 4%. The most critical factor affecting the results was the pH that should be adjusted to be in the range of 8 ± 0.2. Ruggedness was also tested by applying the method to the assay of PXT using the same operational conditions but using two different instruments at two different laboratories and different elapsed time. Results obtained from lab-to-lab and day-to-day variations were reproducible, as the RSD did not exceed 4%.

### Applications of the method

It is evident from the above-mentioned results that the proposed method gave satisfactory results with PXT in its bulk. Thus its pharmaceutical dosage forms (tablets) were subjected to the analysis of their PXT contents by the proposed and the official [6] methods. The label claim percentage was 100.2 ± 1.61% (Table 5). This result was compared with that obtained from the official method by statistical analysis with respect to the accuracy (by t-test) and precision (by F-test). No significant differences were found between the calculated and theoretical values of t-and F-tests at 95% confidence level proving similar accuracy and precision in the determination of PXT by both methods.

Because of the high sensitivity of the proposed method, it was worthy us to check its applicability to the determination of PXT in spiked human plasma. The maximum plasma level of PXT after normal daily doses was found to be in the range of 10–30 ng ml^−1^ [23], which could be assessed by the proposed method using small volume (2 ml) of plasma samples. The high sensitivity of the proposed method allowed the determination of PXT in spiked human plasma. The extraction procedure described by Zainaghi et al. [9] was adopted here. To avoid interferences of water-soluble biogenic amines, the extraction procedures should be carried out before carrying out the reaction between PXT and NBD-Cl. The results were satisfactorily accurate and precise as the recovery was 97.4%–102.2% with RSD less than 3% (Table 6).

## Conclusions

New spectrofluorimetric method for the determination of PXT has been successfully developed and validated. The method involved simple derivatization of PXT with NBD-Cl reagent, and subsequent measuring the fluorescence intensity of the fluorescent reaction product. The proposed method is specific, accurate, reproducible, and highly sensitive to be applied on the analysis of tablets as well as the plasma samples. Furthermore, the analysis is relied on a simple apparatus, thus the proposed method is suitable for routine analysis of PXT in quality control and clinical laboratories.

## Figures and Tables

**Table 1:** Optimization of variables affecting the reaction of PXT with NBD-Cl.

**Variable**	**Studied range**	**Optimum condition**
NBD-Cl (%, w/v)	0.01–0.5	0.2
pH	5–9.5	8 ± 0.2
Temperature (°C)	25–70	40
Time (min)	5–60	30
HCl (M)	0.02–0.5	0.1
Solvent	Different^a^	Acetone
Stability of PXT-NBD (min)	10–60	60

^a^Solvents tested: methanol, ethanol, isopropanol, acetone, and acetonitrile.

^b^The stability of the PXT-NBD was studied after dilution of the reaction solution.

**Table 2:** Statistical parameters for the determination of PXT by the proposed spectrofluorimetric method based on its reaction with NBD-Cl.

**Parameter**	**Value**
λ_ex_ (nm)	490
λ_em_ (nm)	545
Linear range (ng ml^−1^)	80–800
Intercept	5.3550
SD of intercept	1.7646
Slope	0.2304
SD of slope	0.0037
Correlation coefficient (r)	0.9993
LOD (ng ml^−1^)	25
LOQ (ng ml^−1^)	77

**Table 3:** Recovery studies for determination of PXT by the proposed spectrofluorimetric method based on its reaction with NBD-Cl.

**Added (ng ml^−1^)**	**Recovery (% ± SD)^a^**
100	100.2 ± 1.05
150	97.8 ± 1.85
200	99.4 ± 1.28
250	98.4 ± 1.04
300	101.2 ± 1.34

^a^Values are mean of three determinations.

**Table 4:** Analysis of PXT in presence of common excipients by the proposed spectrofluorimetric method.

**Excipient**	**Recovery (% ± SD)^a^**
Starch (50)^b^	102.36 ± 1.82
Glucose (10)	98.76 ± 1.59
Lactose (10)	99.52 ± 2.04
Acacia (10)	101.49 ± 2.15
Talc (5)	103.02 ± 1.95
MS^c^ (10)	99.01 ± 1.76
Average ± SD	100.69 ± 1.83

^a^Values are mean of three determinations.

^b^Figures in parenthesis are the amounts in mg added per 50 mg of PXT.

^c^MS = Magnesium stearate.

**Table 5:** Analysis of PXT-containing-tablets by the proposed and the official methods.

**Dosage form**	**Recovery (% ± RSD)^a^**			
	**Proposed**	**Official^b^**	**t-value^c^**	**F-value^c^**
Seroxate tablets	100.2 ± 1.61	99.6 ± 1.22	2.63	1.74

^a^Values are mean of 5 determinations.

^b^Reference 6.

^c^The tabulated values of t- and F- at 95% confidence limit are 2.78 and 6.39, respectively

**Table 6:** Analysis of PXT spiked in plasma samples by the proposed spectrofluorimetric method.

**Nominal conc. (ng ml^−1^)**	**Measured conc. (ng ml^−1^)**	**Recovery (% ± RSD)**
30	29.4	98.0 ± 2.85
80	77.9	97.4 ± 2.41
120	117.0	97.5 ± 1.85
240	245.2	102.2 ± 2.97
500	490.5	98.1 ± 1.56
